# Contrast-enhanced paravertebrogram to confirm paravertebral catheter position in elective thoracic surgery: a proof of concept study

**DOI:** 10.1007/s00464-020-08087-1

**Published:** 2020-10-28

**Authors:** Fredrik Klevebro, Madhan Kumar Kuppusamy, Shiwei Han, Sara Nikravan, Joseph M. Neal, Wyndam Strodtbeck, David L. Coy, Daniel Warren, Michal Hubka, Neil Hanson, Donald E. Low

**Affiliations:** 1 Department of Thoracic Surgery and Thoracic Oncology, Virginia Mason Medical Center, 1100 Ninth Avenue, Seattle, WA 98101 USA; 2 CLINTEC, Karolinska Institutet, Stockholm, Sweden; 3 Department of Anesthesiology, Virginia Mason Medical Center, Seattle, USA; 4 Department of Radiology, Virginia Mason Medical Center, Seattle, USA

**Keywords:** Paravertebral pain catheters, Postoperative pain treatment, Thoracic surgery, Paravertebrogram

## Abstract

**Background:**

Paravertebral pain catheters have been shown to be equally effective as epidural pain catheters for postoperative analgesia after thoracic surgery with the possible additional benefit of less hemodynamic effect. However, a methodology for verifying correct paravertebral catheter placement has not been tested or objectively confirmed in previous studies. The aim of the current study was to describe a technique to confirm the correct position of a paravertebral pain catheter using a contrast-enhanced paravertebrogram.

**Methods:**

A retrospective cohort proof of concept study was performed including 10 consecutive patients undergoing elective thoracic surgery with radiographic contrast-enhanced confirmation of intraoperative paravertebral catheter placement (paravertebrogram).

**Results:**

The results of the paravertebrograms, which were done in the operating room at the end of the procedure, verified correct paravertebral catheter placement in 10 of 10 patients. The radiographs documented dissemination of local anesthetic within the paravertebral space.

**Conclusion:**

This proof of concept study demonstrated that a contrast-enhanced paravertebrogram could be used in conjunction with standard postoperative chest radiography to add valuable information for the assessment of paravertebral catheter placement. This technique has the potential to increase the accuracy and efficiency of postoperative analgesia, and to set a quality standard for future studies of paravertebral pain catheters.

Enhanced recovery programs after surgery (ERAS) typically include restrictive fluid protocols, regional anesthetic techniques, and standards for early mobilization, all of which can potentially contribute to postoperative hypotension [1, 2]. A stable postoperative hemodynamic environment ensures appropriate splanchnic and cardiac perfusion, thereby decreasing the risk for postoperative complications including myocardial ischemia and anastomotic leak [3]. Although epidural pain catheters are used widely for analgesia after thoracic and abdominal procedures, they typically induce peripheral vasodilatation which can result in significant perioperative hypotension with the potential to impact postoperative morbidity and mortality [4–6].

Furthermore, epidural pain catheters are misplaced in up to 50% [7–12] of patients. Radiographic contrast confirmation of correct epidural pain catheter position can assist troubleshooting of unsatisfactory epidural analgesia by helping to guide the need for catheter replacement or institution of an alternative analgesia technique.

Paravertebral pain catheters have seen increasing application [13] as an alternative to epidural analgesia and have been shown to be effective in patients undergoing thoracotomy and esophagectomy [14, 15]. However, in contrast to using epidurograms to confirm proper placement of epidural catheters, there are no reports of using paravertebrograms for the same purpose. Thus, the incidence and impact of suboptimal placement of paravertebral catheters have not been taken into account in previous studies. The aim of the current proof of concept study was to investigate whether contrast-enhanced radiography (paravertebrogram) can confirm correct location and paravertebral contrast spread prior to leaving the operating room.

## Method

After approval from the institutional research board (Benaroya Research Institute, Virginia Mason Medical Center, Seattle, Washington IRB 19-035), 10 consecutive patients undergoing elective thoracoscopic surgery between January 2019 and July 2019 were identified. Subjects in this retrospective cohort included patients in whom a paravertebral pain catheter was inserted intraoperatively by the surgeon for postoperative analgesia. Radiographic contrast was used to confirm both the vertebral level and correct position of the paravertebral catheter upon completion of the procedure.

### Paravertebral pain catheter insertion

The attending surgeon (MK) inserted the paravertebral pain catheter at the completion of the thoracoscopic procedure with deflated lung. Insertion was typically performed at the thoracic vertebral level T4–T8 appropriate for the thoracic incision using a 17G Tuohy blunt needle (Braun product code: 4512588) in lateral decubitus position. Under direct thoracoscopic visualization, appropriate vertebral level for intended anesthetic infiltration was based on the intercostal incisions relevant for the procedure. The needle puncture of the skin was done near the angle of the rib, 7 to 10 cm lateral from the midline/spinous process, at a perpendicular angle to the selected rib/intercostal space but tangential to posterior parietal pleural surface curvature while simultaneously visualizing the pleura covering the paravertebral space. The Tuohy needle was inserted through the skin in the intercostal space about 7 to 10 cm from the posterior midline (spinous process palpated over the sterile drape) on the top border of the lower rib. Near the posterior most aspect, the neurovascular bundle can be often seen in the intercostal space rather than behind the ribs. Therefore, the needle should be aimed perpendicular to the chest wall at the entry point until the bevel of the needle facing away from the pleura and seen inside the chest in the intercostal space bulging behind the pleura lateral to the sympathetic chain; then needle tip direction can be slightly changed to keep it behind the pleura, avoiding the neurovascular structures and allowing advancement without puncturing the pleura until the tip reaches just medial to the sympathetic chain.

Although it is very rare, if there is any bleeding at this stage from inadvertent intercostal vessel injury by the needle tip, it can be stopped by simple pressure at the site of bleeding or proximal to it using blunt instruments from inside the chest after withdrawing the needle slightly. Once the bleeding is controlled, same needle entry can be advanced to reach paravertebral space or in case of pleural puncture if needed, choose an intercostal space above or below for needle reinsertion.

Once the needle tip reached just medial to the sympathetic chain, the bevel of the needle was tilted toward cephalic and caudal directions, and 10–30 ml of 0.9% sterile saline was infiltrated, which opened the paravertebral space and significantly extended anesthetic agent spread and block coverage. The tip of the needle was kept behind the semitransparent pleura within the saline-infiltrated paravertebral space, which at this point was bulging in the caudo-cephalic direction for at least 5 to 6 intercostal spaces.

While the bevel of the needle was facing in the caudal direction, a 19G blunt tip catheter with side holes (Braun product code 451 3258 C) was inserted. The length of catheter insertion varied based on the angle with which the paravertebral space was reached, tissue depth from the skin to paraspinal needle entry point, and the widening of paravertebral space that allows the catheter to reach further or coil. As the tip of the catheter was seen emerging from the bevel, the needle tip was kept stable. This enables the needle to be rotated slightly as needed to avoid the catheter tip puncturing of the pleura and also to help guiding the catheter reach one or two spaces towards cephalic direction. At least 5–7 cm length of catheter should be advanced beyond the needle tip into the paravertebral space. It is recommended to use a catheter with side holes and blunt tip, which helps wider distribution of the block, reduces catheter blockage, and ensures that the tip of the catheter travels easily in the intended direction. After removing the Tuohy needle with the catheter still in the paravertebral space, it is fixed to the skin using standard techniques and/or kits available for epidural catheters. Finally, after attaching a Luer-lock adaptor to the catheter, 20 ml of 0.5% Bupivacaine was injected resulting in local anesthetic spread within the paravertebral space, a further 1 or 2 vertebral levels in both cranio-caudal directions.

### Paravertebrogram

Radiographic confirmation of the paravertebral catheter placement and extent of dissemination of local anesthetic was performed in conjunction with our institutional standard postoperative anteroposterior chest X-ray immediately following completion of the case. Using an aseptic technique, 5 ml of sterile preservative-free Iopamidol 61% (Isovue-M-300, Bracco Imaging, Monroe Township, USA) was injected through the paravertebral catheter. The surgeon and the anesthesiology team reviewed the paravertebrogram before leaving the operating room. Images were assessed for (i) vertebral level of paravertebral catheter tip; (ii) evidence of contrast within paravertebral space; (iii) extent of vertical contrast spread (number of vertebral levels above and below catheter tip); and (iv) lateral spread of contrast (midline, right, or left). In cases where there was uncertainty regarding the findings of a paravertebrogram, one of three senior members of the anesthesiology pain service was consulted. A negative paravertebrogram was interpreted as a misplaced catheter, and, according to protocol, the patient would have received an alternative postoperative pain analgesic. The anesthesiology pain service followed patients to determine the requirement for additional clinical interventions based on patient’s clinical status. For the purpose of this study, a senior radiologist (DC) retrospectively reviewed all images.

### Study outcome

The aim of this study was to investigate if correct positioning of paravertebral pain catheters can be confirmed with the use of contrast radiography in conjunction with the mandatory postoperative radiography exam. Secondary outcomes included monitoring for adverse events during the length of hospital stay, i.e., puncture of the pleura, defined as a spread of contrast in the thorax on radiography or visual detection of fluid leak, limited spread of contrast indicating that the paravertebral space was not opened, paravertebral bleeding, infection, allergic reaction, failed analgesia hemodynamic instability, or postoperative complications.

### Data collection and statistical analysis

Patient data were retrieved from electronic medical records and included details of surgery and paravertebral pain catheter placement, adverse events, and result and interpretation of paravertebrogram.

## Results

Ten patients who underwent a thoracoscopic procedure and received paravertebral pain catheters for postoperative pain were analyzed retrospectively. All catheters were successfully placed on the first attempt. Placement of the catheter increased the length of time under general anesthesia by approximately 5–7 min. Patient and treatment characteristics are presented in Table 1.

**Table 1:** Patient and treatment characteristics and postoperative outcomes

Mean age in years (range)	64 (29–78)
Female, *n* (%)	7 (70.0)
Male, *n* (%)	3 (30.0)
Mean Body Mass Index (range)	27.3 (19.1–42.1)
Malignant disease, %	8 (80.0)
Mean Charlson Comorbidity Index with age (range)	4 (0–6)
American Society for Anesthesiology Score	
II, n (%)	5 (50.0)
III, *n* (%)	5 (50.0)
Surgical procedure	
Lobectomy, *n* (%)	4 (40.0)
Wedge resection, *n* (%)	4 (40.0)
Bullectomy, *n* (%)	2 (20.0)
Surgical technique	
Multi-port thoracoscopy, *n* (%)	4 (40.0)
Single-port thoracoscopy, *n* (%)	6 (60.0)
Mean operative time in hours (range)	3.5 (2.5–5.0)
Postoperative outcomes	
Postoperative complications, *n* (%)	1 (10.0)
Pneumonia, *n* (%)	1 (10.0)
Clavien-Dindo Score	II (One patient)
Mean Hospital Length of Stay in days (range)	2.7 (1–7)
Mean postoperative pain score^a^ (IQR)	5.6 (4–7)
Paravertebrogram results	
Correct paravertebral catheter placement, *n* (%)	10 (100.0)
Mean number of vertebral levels seen with vertical contrast spread (range)	4.5 (4–6)

^a^Calculated using all values during first 24 postoperative hours

**Fig. 1:**
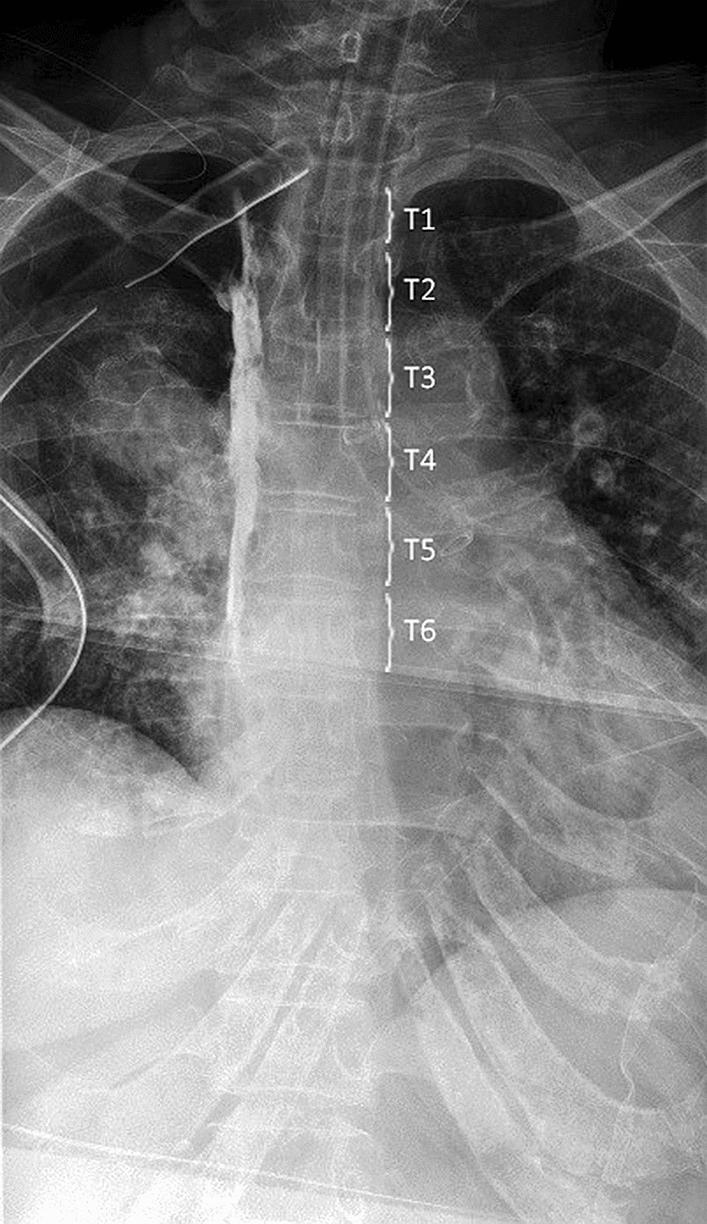
Example of a paravertebrogram showing contrast in the paravertebral space on the right side. In this patient, a single-port right upper lobectomy was performed through a one-inch incision in the 5th intercostal space. The catheter was placed through the 4th paravertebral space

Paravertebrogram was performed in 10 consecutive patients with the use of contrast-enhanced anteroposterior spine radiography. No adverse events related to the paravertebrogram were detected. One patient had postoperative pneumonia resulting in Clavien-Dindo score II. The surgical and anesthesiology team reviewed all images before leaving the operating room and determined that the catheter was identified in correct position within the paravertebral space in all 10 patients (100%, Fig. 1). The contrast on average spreads across 4.5 vertebral levels. The radiologist reviewed all images and confirmed the correct interpretation of the paravertebrograms. Criteria for correct placement of the catheter included dye in a confined compartment approximately 2–3 cm wide adjacent to the spine. The medial border represents the vertebra and is visible as a straight line; the lateral border shows indentations of the ribs and nerve bundles.

## Discussion

This proof of concept study is the first to demonstrate the feasibility and efficacy of confirming paravertebral pain catheter position with the use of an intraoperative paravertebrogram. This technique provides an opportunity for objective confirmation of paravertebral pain catheters, which gives valuable information relevant to immediate postoperative pain management and, if applied in clinical research, can increase the quality of paravertebral pain catheter studies by confirming proper catheter placement, thereby facilitating opportunity to adjust or replace the catheter prior to leaving the operating room. Paravertebrogram can be performed in conjunction with mandatory postoperative radiography, which makes the exam time efficient and adds only the cost of contrast dye and a few minutes of time in the operating room. The added risk for the patient from contrast injection is small. What is more, the results of the study demonstrated that the interpretation of paravertebrograms may not require the assistance of a radiologist, and this expense can be offset with less additional postoperative assessments and clinical reviews associated with the uncertainty of catheter position.

A randomized controlled trial showed that paravertebral pain catheters were more effective than epidural pain catheters for controlling post-thoracotomy pain and maintaining pulmonary function, while being associated with fewer respiratory complications, and reduced levels of postoperative nausea and hypotension [16]. A subsequent meta-analysis showed that paravertebral pain catheters were also associated with significantly less urinary retention, nausea and vomiting, postoperative hypotension, and rates of failed blocks yet with similar postoperative pain scores compared to epidural pain catheters [17]. A weakness of previous comparative studies of paravertebral vs. epidural pain catheters is that correct catheter placement has not been objectively confirmed. Epidural pain catheters have been shown to be misplaced in 14–43% of patients, and there are no previous studies about misplacement risk for paravertebral pain catheters which make interpretation of study results problematic [7–11].

The current study has limitations that need to be recognized. The limited sample size of this proof of concept study warrants further investigation. Most importantly, our paravertebrograms were performed utilizing a single anteroposterior radiograph. We acknowledge that under normal circumstances, two planes of view improve the assessment of accurate catheter placement. A lateral view was impossible within our study design because it added an additional, nonstandard aspect to the routine care of this patient cohort. Moreover, intraoperative lateral thoracic films are typically not of high quality because of patient position and sterility issues. Further investigation will be required to assess better methods for obtaining clinically useful lateral or oblique angles. Of note, intentional misplacement of a paravertebral catheter for the purpose of illustrating a negative study was considered unethical. Nevertheless, the current technique provides valuable information. First, placing paravertebral catheters under thoracoscopic guidance and observing the expected expansion of the paravertebral space upon contrast injection strongly suggest proper catheter siting. Second, all of our AP images were consistent with paravertebral catheter placement, and none were obscured by the spine, thus providing “in the moment” information even in the absence of a confirmatory (but impractical) lateral view. Third, a paravertebrogram had not been observed on AP image, and then further lateral imaging would be moot.

In conclusion, this proof of concept study describes successful use of an objective technique for evaluating the position of a paravertebral pain catheter using a paravertebrogram obtained in conjunction with a routine postoperative chest radiograph. If our findings are confirmed by subsequent clinical studies, the use of a paravertebrogram might aid in clinical management of paravertebral pain catheters. Importantly, utilizing paravertebrograms and epidurograms should improve the quality of future comparative investigations of the techniques by objectively confirming proper catheter placement.

## Abbreviation

ERAS: Enhanced recovery programs after surgery
